# Childhood Adversity Is Associated with Adult Theory of Mind and Social Affiliation, but Not Face Processing

**DOI:** 10.1371/journal.pone.0129612

**Published:** 2015-06-12

**Authors:** Laura Germine, Erin C. Dunn, Katie A. McLaughlin, Jordan W. Smoller

**Affiliations:** 1 Psychiatric and Neurodevelopmental Genetics Unit, Massachusetts General Hospital, Boston, Massachusetts, United States of America; 2 Center for Human Genetic Research, Massachusetts General Hospital, Boston, Massachusetts, United States of America; 3 Department of Psychiatry, Harvard Medical School, Boston, Massachusetts, United States of America; 4 Department of Psychology, Harvard University, Cambridge, Massachusetts, United States of America; 5 Department of Psychology, University of Washington, Seattle, Washington, United States of America; University of Akron, UNITED STATES

## Abstract

People vary substantially in their ability to acquire and maintain social ties. Here, we use a combined epidemiological and individual differences approach to understand the childhood roots of adult social cognitive functioning. We assessed exposure to 25 forms of traumatic childhood experiences in over 5000 adults, along with measures of face discrimination, face memory, theory of mind, social motivation, and social support. Retrospectively-reported experiences of parental maltreatment in childhood (particularly physical abuse) were the most broadly and robustly associated with adult variations in theory of mind, social motivation, and social support. Adult variations in face discrimination and face memory, on the other hand, were not significantly associated with exposure to childhood adversity. Our findings indicate domains of social cognition that may be particularly vulnerable to the effects of adverse childhood environments, and suggest mechanisms whereby environmental factors might influence the development of social abilities.

## Introduction

Human beings are social animals built to interact and acquire information about the world through others, seek social affiliation, and exist in social communities. Deficits in the ability to develop and maintain social relationships are a key component of many mental disorders [1] and social support is a significant predictor of physical health and mortality [2]. But what makes social interaction pleasurable and easy for one person, yet challenging and unrewarding for another? Understanding the factors that drive variations in social cognitive functioning are key to understanding psychological health and well being.

Research focused on cognitive and social cognitive outcomes of childhood adversity have indicated that some adversities affect cognitive development very broadly [3] whereas other childhood adversities are associated with more specific aspects of social cognitive development [4]. For example, severe childhood neglect (e.g. institutionalization) has a lasting impact on many aspects of cognitive functioning [3], including aspects of social cognition such as face emotion perception [5,6] and social attachment [7]. Physical abuse, on the other hand, is associated with subtle biases in emotion perception, but not with deficits in recognition of basic emotions [4]. The consequences of childhood adversity on social cognitive functioning thus appear to vary both by social cognitive domain and adversity type [8], with evidence that severe neglect is associated with deficits in basic emotion recognition whereas physical abuse is associated with more specific biases in emotion judgments [6]. These previous findings indicate that both severe neglect and physical abuse might alter how children interpret the thoughts and feelings of other people (also known as “theory of mind”), a core social-cognitive ability that contributes to how well a person is able to successfully navigate social interactions and relationships.

Large-scale psychiatric epidemiology studies indicate that experiences of childhood adversity also have a substantial negative impact on adult mental health [9–12]. The impact of childhood adversity on mental health, however, appears to be relatively nonspecific -- childhood adversities increase risk for virtually all commonly occurring mental disorders [10,11].

These two literatures provide contrasting views on how adversity might impact psychological development. Research on individual differences in social cognition suggests relatively specific relationships between certain types of childhood adversity and social cognitive functioning [4,6]. Mental health research, on the other hand, suggests lack of specificity between exposure to major childhood adversities and mental disorders [10,11]. Do these differences reflect true differences between psychiatric vs. social cognitive outcomes, or potential differences in methodology? Foundational work in psychiatric epidemiology has relied on the assessment of many different types of childhood adversity exposure in very large, unselected samples [10,11]. Research in social cognition and adversity, on the other hand, has tended to rely on smaller, more targeted samples with specific types of adversity exposure [3,4,6]. No study has yet combined the two approaches, to map the landscape of associations between common forms of childhood adversity and objectively measured social cognitive outcomes in very large samples.

In this study, we sought to understand the relationship between childhood adversity and social cognitive functioning in adulthood by combining the methods of psychiatric epidemiology and individual differences research. Findings from psychiatric epidemiology have shown that common forms of childhood adversity are related to increased risk of mental disorders *in adults*. This suggests that childhood adversity leaves lasting impacts on information processing that persist beyond childhood. However, as most studies of social cognition and childhood adversity are conducted in developing children experiencing the most severe cases of adversity, it is unclear how findings from these studies might explain the relationship between psychiatric vulnerability in adulthood and early adversity in the broader population. Our goal was to extend previous findings in childhood adversity and social cognitive development by investigating the relationship between social cognition and a broader range of relatively common childhood adversities, in a large sample of adult age participants. An appreciation of the relationship between common forms of childhood adversity and adult age social cognition and affiliation will suggest mechanisms that might link early life stress with lasting differences in social functioning and ultimately mental health. With this in mind, our primary aims were to (1) identify childhood environments associated with poor social-cognitive functioning in adulthood, and (2) determine which domains of social cognition are most sensitive to childhood stress and adversity.

Based on previous literature, we hypothesized that physical abuse would be linked with biases in mental state inferencing or theory of mind, whereas childhood neglect would be related to both reduced theory of mind ability and reduced ability to discriminate basic facial emotions [5,6]. We further hypothesized that face identity recognition would be minimally impacted by childhood adversity experiences. Previous work has indicated that face identity recognition ability is based almost entirely on heritable factors, with little to no role for shared or unshared environment [13] and that face processing is relatively unaffected by childhood trauma and deprivation [8,14,15]. Finally, we hypothesized that the same childhood adversity experiences that impact social cognitive ability would also be associated with reduced social motivation and social support. In other words, we hypothesized that childhood adversity experiences linked with differences in adult social cognition would also be associated with everyday differences in social affiliation that may be proximally related to poorer mental and physical health [2].

One critical barrier to the type of comprehensive, large-scale analysis proposed here are the sample sizes needed to assess a wide range of childhood adversities along with objectively measured social cognitive outcomes, while maintaining the power necessary to overcome issues of multiple comparisons. To overcome this challenge, we took advantage of well-validated web recruitment and assessment methods through TestMyBrain.org. TestMyBrain.org is a citizen science website that uses crowdsourcing methodologies to collect large sample datasets for behavioral experiments. Data from TestMyBrain.org has previously been shown to be comparable in quality to data collected in the lab [16].

## Materials and Methods

### 2.1 Overview

We selected 25 adversities from previous psychiatric epidemiology studies that have linked childhood adversities with adult mental disorders. These included experiences of physical abuse, sexual abuse, neglect, and parent loss as well as parental behaviors such as drug abuse, alcoholism, and criminal activities [10–12]. Each participant completed 1–2 measures of social cognitive functioning and/or social affiliation followed by the TestMyBrain Childhood Experiences Questionnaire. These included objective measures of face discrimination for emotion and identity [17,18], face memory [19], theory of mind (or mental state inferencing) [20], as well as self-report measures of social motivation [21,22] and social support [23]. Web links to all measures (identical to those viewed by participants) are included below the descriptions of each measure.

### 2.2 Childhood Adversity Experiences

We assessed childhood adversity experiences by asking participants to answer questions about experiences they had from the time they were born until age 18. The TestMyBrain Childhood Experiences Questionnaire is adapted from the Adverse Childhood Experiences Scale [24], Conflict Tactics Scale [25], and Composite International Diagnostic Interview [26]. Participants reported whether or not they had been exposed to the following adversities: parent death, parent divorce, institutionalization or foster care, parental alcoholism, parental drug abuse, parental mental illness, parental suicide, or parental imprisonment; sexual abuse and/or rape. Given that we were ascertaining adversity exposure through self-report and could not ask follow-up questions, we adopted a strict threshold for classifying an individual as “exposed”. As there were many more unexposed individuals than exposed individuals for nearly all childhood adversities that we assessed, this decision was made to minimize noise due to misclassification in the exposed group. Thus, participants answering “yes” were coded as exposed, whereas participants answering “no”, “I don’t know”, or “I’d rather not say” were coded as not exposed. Participants also reported the frequency (never/sometimes/rarely/often) of the following adversities: parental criminal behavior, domestic violence (two items), verbal abuse, physical abuse (three items), fear of abuse (one item), hunger due to poverty, and parental neglect (five items). Similarly, participants were coded as exposed if they had the experience “sometimes” or “often” and not exposed for all other response options (e.g., “rarely”, “never”, “I don’t know” or “I’d rather not say”). The full childhood experiences questionnaire can be found in S1 Table of supplementary materials and at this link: http://www.testmybrain.org/tests/childhood_adversity.html

### 2.3 Social Cognition and Social Affiliation

**Fig 1:**
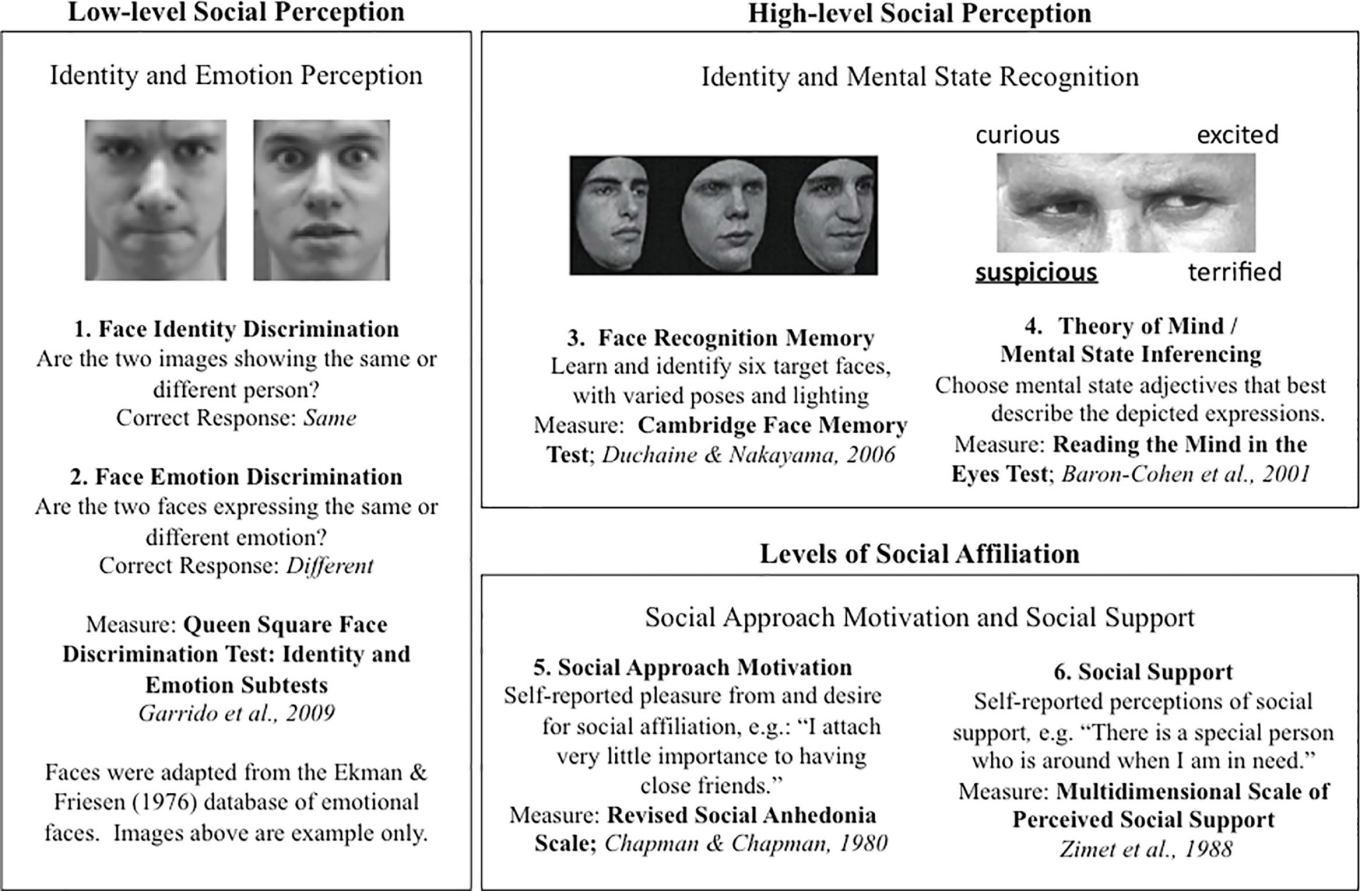
Measures of social cognition and social affiliation. All images are for illustrative purposes only and do not reflect items appearing in the actual test measures. Measures (1) and (2) used images adapted from the Ekman and Friesen database [40]. Illustration images adapted from CC-BY licensed images originally appearing in Skelly and Decety, 2012 [41]. Illustration image for measure (4) reprinted from Duchaine & Nakayama 2006 [19] under a CC BY license, with permission from Bradley C. Duchaine, original copyright 2006. Reprinted with permission. The illustration image for measure (4) was adapted from a public domain (CC0) photograph: http://www.pdpics.com/photo/3564-drum-seller/

Four measures of social cognition and two measures of social affiliation were included in this study. There is a wide range of possible measures of social cognitive functioning, but for the purposes of this study we focused on well-validated measures of high-level social inferencing, social recognition, and the regulation of social behavior. This included measures of theory of mind (inferring mental states), face identity discrimination, face emotion discrimination, self-reported social motivation, and self-reported social support. Aspects of social cognition not assessed included the ability to acquire social/affective responses, embodied aspects of social cognition, and context-specific regulation of social-emotional responses [27]. Fig 1 gives example stimuli, trials, and/or items for each of the six measures.

#### 2.3.1 Face Discrimination

To measure face discrimination, we administered the emotion and identity subtests of the Queen Square Face Discrimination Test (QFDT emotion and QFDT identity) [17,18]. In this task, two female faces are shown sequentially for 500 milliseconds per face. In the emotion subtest, the participant decides if the faces express the same or different basic emotion (happy, sad, fearful, angry, disgusted, or surprised). In the identity subtest, the participant decides if the faces are the same person or two different people. The two subtests are matched in task, length, difficulty, and stimuli. The QFDT emotion and identity subtests are behaviorally and neurally dissociable, tapping into different aspects of face discrimination [17,18,28,29]. For each test, a participant’s score is the proportion correct out of 72 trials. http://www.testmybrain.org/tests/qfdt/emotion.html http://www.testmybrain.org/tests/qfdt/identity.html

#### 2.3.2 Face Recognition Memory

Face memory depends on both intact facial perception and the ability to encode and retrieve face information. We measured face memory using the Cambridge Face Memory Test [19]. In this test, participants learn and then recognize six male faces in conditions of increasing difficulty (e.g. differing viewpoints, lighting, or with visual noise added). The participant’s score is the proportion correct out of 72 trials.

This test is highly sensitive to impairment [19], taps into face memory independent of general memory ability [30], and variations in performance are based almost entirely on heritable factors [13]. http://www.testmybrain.org/tests/cambridge_face_memory_test/

#### 2.3.3 Theory of Mind / Mental State Inferencing

Theory of mind is the ability to infer the mental state of another person. We measured theory of mind using the Reading the Mind in the Eyes test [20]. In this test, participants see 36 images of the eye regions of faces and decide which of four adjectives best describes the mental state of each pair of eyes. This test is highly sensitive to social cognitive impairment and low scores have been linked to extensive real world difficulties in social interaction and understanding [20]. http://www.testmybrain.org/tests/mind_in_eyes/

#### 2.3.4 Social Motivation/Pleasure

We measured social motivation using the Revised Social Anhedonia Scale [21]. This scale comprises 40 items assessing how much a person seeks and enjoys social interactions. High levels of social anhedonia (low social pleasure/motivation) predict the development of psychiatric disorders [22]. http://www.testmybrain.org/tests/anhedonia.html

#### 2.3.5 Perceived Social Support

We measured participant’s perceptions of current social support from friends and family using the 12-item Multidimensional Scale of Perceived Social Support [23]. http://www.testmybrain.org/tests/mspss.html

### 2.4 Web Administration and Sampling

All participants were anonymous visitors to the website TestMyBrain.org who visited the site between December 21, 2012 and December 2, 2013. TestMyBrain is a citizen science website where people participate in experiments in exchange for feedback on their performance. No explicit advertising or recruitment is conducted. Participants come through search engines and links typically generated by previous participants. The quality of data collected on TestMyBrain is comparable to data collected using traditional methods, even for challenging tests that rely on accurate visual perception of social information [16]. All participants who took part in this experiment clicked on a link for “The Social Mind and Life Experiences”, with no reference to childhood adversity.

Each of the social functioning measures described below was available on TestMyBrain at a different time, to keep each test battery as brief as possible (20 minutes or less) and minimize participant attrition. Each battery collected data for two months, the estimated time needed to obtain 1,000 participants after exclusions (see next paragraph) and achieve 90% power to detect associations of a magnitude r > 0.1. An initial pilot battery included just the Reading the Mind in the Eyes test and the childhood experiences questionnaire. In the next battery, we included both the Reading the Mind in the Eyes test and the Multidimensional Scale of Social Support. The next battery had the Cambridge Face Memory Test and the Revised Social Anhedonia Scale. The final battery included the two subtests of the Queen Square Face Discrimination Test. We report results from all batteries and phases, including data collected using the Reading the Mind in the Eyes in the pilot phase of this protocol as no changes to the basic protocol were made. Variations in sample size reflect variations in website traffic and participant demographics during the times each battery was available online, as well as inclusion of data from the initial pilot phase.

For each battery, all participants who reported an age of 18 years or older were asked to participate in a childhood experiences questionnaire that would include questions about childhood trauma/abuse or complete a different questionnaire with less sensitive questions about their everyday experiences. No personally identifying information was collected. Participants provided informed consent by electronically signing a form prior to participation. The study and consent procedure was approved by the Harvard University Committee on the Use of Human Subjects in Research (CUHS).

### 2.5 Exclusions

For this study, we sampled adults who had grown up primarily in industrialized, English-speaking countries. Of those who completed the experiment we excluded anyone who: (1) indicated they had participated in the same or similar experiment before (3%), (2) indicated they had technical problems that may have interfered with their responses (6%), (3) reported a gender other than male or female (2%), (4) indicated they used strategies that may be considered cheating (2%), or (5) opted out of the TestMyBrain Childhood Experiences Questionnaire (13%). Our final sample included 5,559 participants. Sample sizes for each social cognition or social functioning measure ranged from 930 to 2,242.

### 2.6 Data Analysis

#### 2.6.1 Primary Analysis: Principal Component Analysis (PCA) of Adversity and Social Cognition/Affiliation

Childhood adversities often occur together (e.g. physical abuse and domestic violence) and it is difficult to say whether any observed associations between childhood adversity and outcome reflect a particular class of experience or to the overall environments in which those experiences occur. Our primary analysis used adversity exposure data across all participants to derive components that would capture the covariance structure of childhood adversities. This analysis allowed us to: (1) compare the particular components derived from our data to the literature on childhood adversity experiences as a way of validating our web-based, crowdsourcing method, (2) account for the covariance structure of the adversity data in our sample, and (3) reduce the number of comparisons made. All adversity data were submitted to a standard principal component analysis (PCA) with varimax rotation of the resulting eigenvector components, using the *principal* function from the *psych* package in R [31].

Resultant component scores were used as predictors of social cognition and affiliation scores using linear regression (1) without any covariates, (2) controlling for age, sex, and race/ethnicity, and (3) controlling for age, sex, race/ethnicity and childhood socioeconomic status as indexed by parental education, relative household income, and participation in government assistance programs. Maternal and paternal education were combined into a single parental education variable equal to the highest known education level of either parent. Linear regression models were estimated using the *lm* function from the *stats* package in R [32].

#### 2.6.2 Secondary Analysis: Individual Adversities and Social Cognition/Affiliation

We also looked at each individual adversity separately as a predictor of scores on each social cognition/affiliation measure. This allowed us to take advantage of the richness of this dataset and identify associations that may have been obscured or unaccounted for by PCA dimension reduction.

We conducted two-tailed independent samples t-tests to compare social cognition/affiliation scores between individuals who experienced and did not experience each adversity, after adjusting for sex, age, and race/ethnicity in social measures. Results are reported in terms of *Cohen’s d*. T-tests were conducted using the *t*.*tests* function from the *stats* package in R [32].

#### 2.6.3 Reporting of Results

All effect sizes are reported regardless of significance along with 95% confidence intervals. Significance was then reported based on (1) p < 0.05 uncorrected, (2) Bonferroni correction for number of predictors for each social cognitive or social functioning outcome, and (3) Bonferroni correction for all comparisons.

#### 2.6.4. Data Availability Statement

All data used in the described analyses are available through FigShare http://figshare.com/articles/Data_from_Germine_et_al_2015_PlosONE_Childhood_adversity_and_social_perception/1425185 and the Open Science Framework: https://osf.io/dih6a/

## Results

### 3.1 Childhood Adversity

Summary characteristics for all subsamples are shown in Table 1. Prevalence estimates for each adversity are shown in the top portion of Fig 1 and ranged from 3% (institutional care) to 44% (verbal abuse). Prevalence estimates broken down by social cognition or social affiliation measure are given in S2 Table of supplementary materials. Using PCA, we derived four components that explained roughly half of the variance in childhood adversity experiences. The first component (explaining 14% of total variance) was related to adversities indexing *parental maltreatment*, including experiences of physical abuse, verbal abuse, and domestic violence. The second component (explaining 12% of total variance) was most related to adversities related to *parental maladjustment*, again including domestic violence, but also criminal activity, divorce, alcoholism, and drug abuse. The third component (explaining 11% of total variance) related to *parental neglect*, such as neglect of parents to provide regular meals, clothing / school supplies, supervision, safety, and medical care. The fourth and final component (explaining 9% of total variance) related to both experiences of *sexual abuse* and *institutional care / foster* care.

**Table 1:** Sample Characteristics.

	Face Emotion Discrimination	Face Identity Discrimination	Face Recognition Memory	Mental State Inferencing	Social Motivation	Social Support	Childhood Adversity
**Number of participants**	**1504**	**1504**	**1706**	**2242**	**1706**	**930**	**5559**
**proportion right-handed**	0.86	0.86	0.87	0.88	0.87	0.87	0.87
**proportion male**	0.31	0.31	0.34	0.34	0.35	0.29	0.33
**Race/ethnicity proportions**							
nonhispanic white	0.74	0.74	0.77	0.74	0.77	0.75	0.75
nonhispanic black	0.02	0.02	0.03	0.04	0.03	0.03	0.03
hispanic	0.07	0.07	0.05	0.06	0.05	0.07	0.06
other race/ethnicity or decline to respond	0.16	0.16	0.15	0.16	0.15	0.16	0.16
**Education (years)**	14.9	14.9	15.1	15	15.1	15	15
**Participant age at testing**							
mean	33.5	33.5	32	32.7	32	31.4	32.5
SD	13.7	13.7	12.6	13.3	12.6	12.8	13.2
**Country of origin**							
US*	0.76	0.76	0.71	0.76	0.71	0.73	0.75
Canada	0.08	0.08	0.08	0.09	0.08	0.1	0.08
UK	0.13	0.13	0.18	0.13	0.18	0.15	0.14
Australia / New Zealand	0.09	0.09	0.05	0.05	0.05	0.05	0.06
Ireland	0.01	0.01	0.02	0.01	0.02	0.01	0.01

All participants who completed any of the six social cognition or social affiliation measures also completed the childhood experiences questionnaire. Different participants completed different social cognition/affiliation measures to minimize the burden of testing for each individual and thus minimize participant attrition. See “Web Administration and Sampling” for details of sampling procedures.

**Fig 2:**
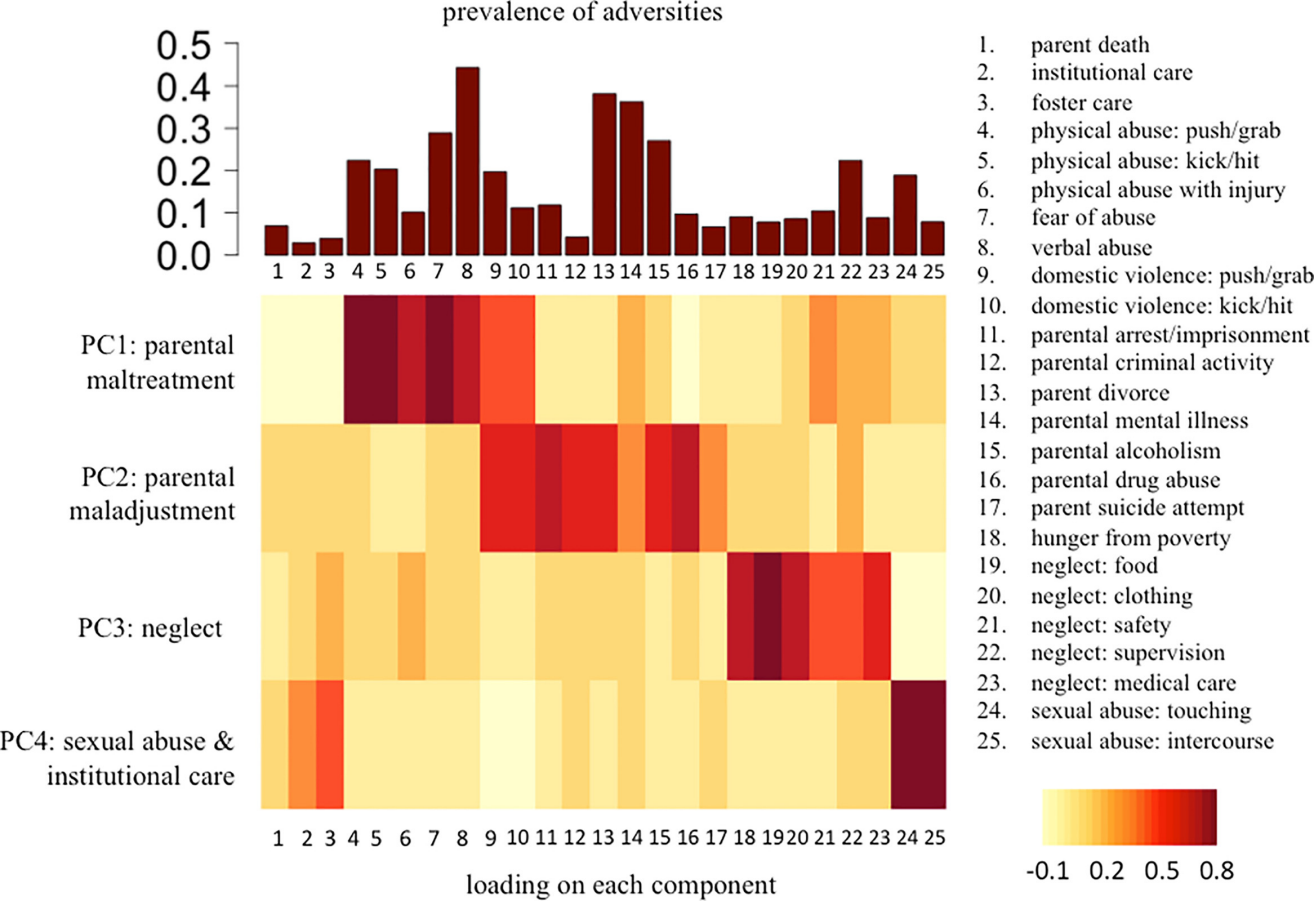
Adversity prevalences and relationships with PCA-derived components. In the top part of the figure, bars represent the number of participants who were coded as exposed to each type of adversity. The bottom of the figure shows a heatmap relating each adversity to a set of four principal components derived from principal components analysis with varimax rotation. Darker shades of red represent higher loading of an adversity on a particular component.

Factor loadings for each adversity on each component are shown in the bottom portion of Fig 2 and in S3 Table of supplementary materials.

### 3.2 Adversity Components and Social Cognition/Affiliation

Summary scores for each social cognitive or social affiliation measure are shown in Table 2.

**Table 2:** Summary scores and internal reliability for each social cognitive or social functioning measure.

	Mean	SD	Reliability
**Face Emotion Discrimination**			
Queen Square Face Discrimination Test: Emotion	0.77	0.08	0.69
**Face Identity Discrimination**			
Queen Square Face Discrimination Test: Identity	0.78	0.09	0.74
**Face Recognition Memory**			
Cambridge Face Memory Test	0.75	0.14	0.9
**Theory of Mind / Mental State Inferencing**			
Reading the Mind in the Eyes	0.7	0.14	0.75
**Social Motivation**			
Revised Social Anhedonia Scale	14.7	7.9	0.88
Range: 0–40			
**Social Support**			
Multidimensional Scale of Perceived Social Support	58.6	15.3	0.92
Range: 11–77			

Scores for measures of social cognition (face emotion discrimination, face identity discrimination, face recognition memory, and theory of mind) are given in terms of mean and standard deviation of proportion correct. Scores for measures of social affiliation (social motivation and social support) are given in terms of mean and standard deviation of total scores, where the range of possible scores is given under the name of each measure. Reliability is reported in terms of Cronbach’s alpha, a measure of internal reliability or consistency.

**Fig 3:**
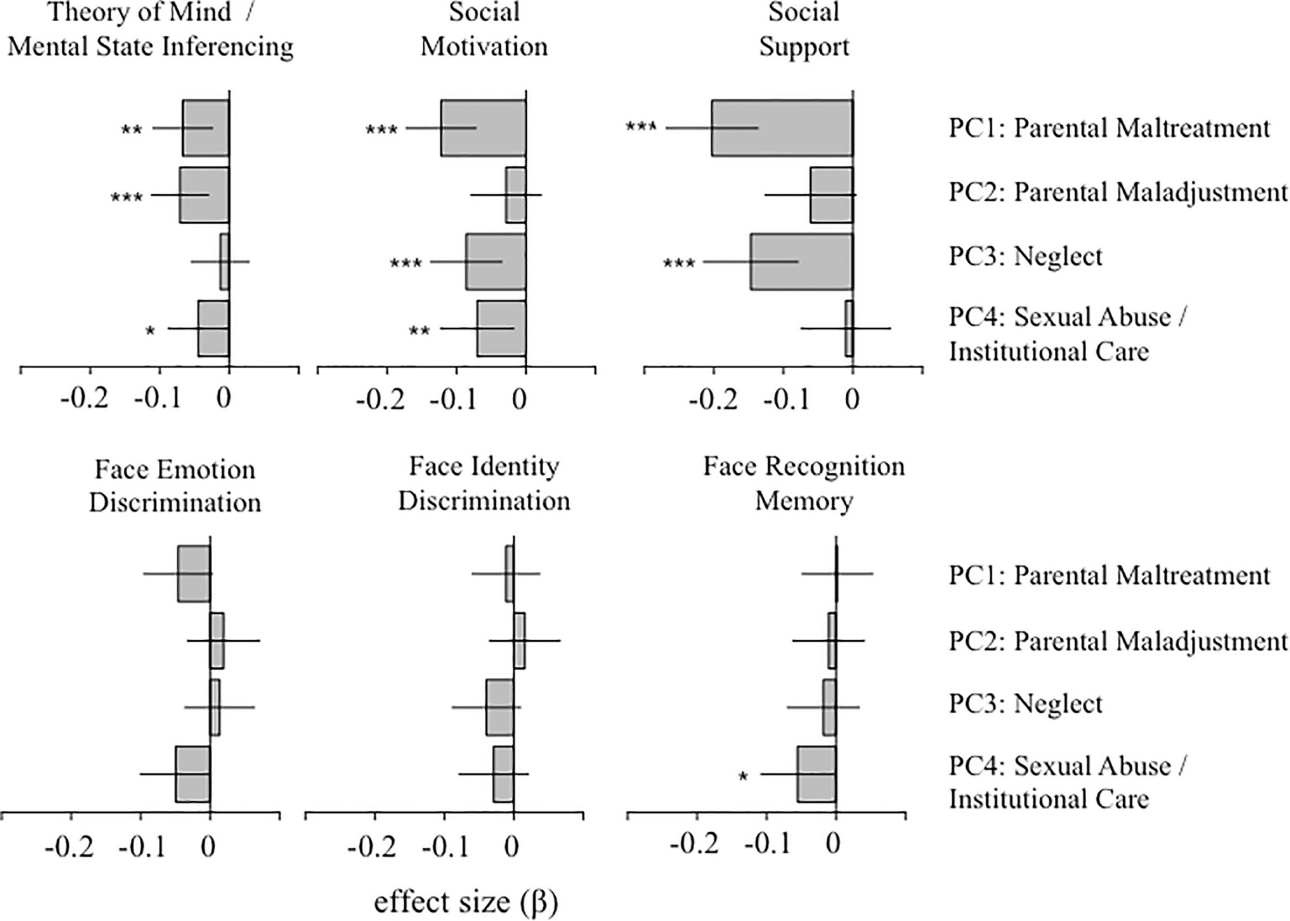
Relationship between PCA-derived adversity components and six domains of social cognition and social affiliation. Results are given in terms of standardized regression coefficients, after controlling for influence of age, sex, and race/ethnicity on each dependent measure. Solid lines give 95% confidence intervals for each effect size estimate. Three asterisks indicate associations that were significant at p < 0.0021 (Bonferroni corrected for all comparisons), two asterisks indicate associations significant at p < 0.0125 (Bonferroni corrected for number of orthogonal comparisons in each model), and one asterisk indicates nominal significance at p < 0.05. Theory of mind ability, social motivation, and social support were all robustly associated with parental maltreatment—with reductions in scores across all three measures. Parental maladjustment was most associated with reduced theory of mind ability, parental neglect with reduced social support in adulthood, and sexual abuse / institutional care with reduced social motivation in adulthood. None of the face discrimination or face recognition memory showed more than nominal associations with childhood adversity.

Results from linear regression using adversity component scores as predictors of social cognition / social affiliation are shown in Fig 3. Results are given in terms of standardized regression coefficients and associated 95% confidence intervals, controlling for covariates. Three levels of statistical significance are indicated: nominal statistical significance (p < 0.05), significance after correcting for number of components per model (4 comparisons; p < 0.0125), and significance after correcting for the number of comparisons made across all six models (24 comparisons; p < 0.0021).

Parental maltreatment (PC1) was robustly associated with theory of mind ability (β = -0.067, p < 0.01), social motivation (β = -0.12, p < 0.0001) and social support (β = -0.20, p < 0.0001). Parental maladjustment (PC2) was only significantly related to theory of mind ability (β = -0.07, p < 0.001). Parental neglect (PC3) was associated with social support (β = -0.15, p < 0.0001) and social motivation (β = -0.086, p < 0.001). Finally, sexual abuse / institutional care (PC4) was only related to social motivation (β = -0.07, p < 0.01). None of the measures of face discrimination or memory (including face emotion discrimination) were significantly associated with experiences of childhood adversity. Controlling for socioeconomic status did not produce any notable changes in the pattern or significance of these results. Coefficients and statistics for all estimated models (with and without covariates) are given in S4 Table of supplementary materials.

### 3.3 Individual Adversities and Social Cognition/Affiliation

**Fig 4:**
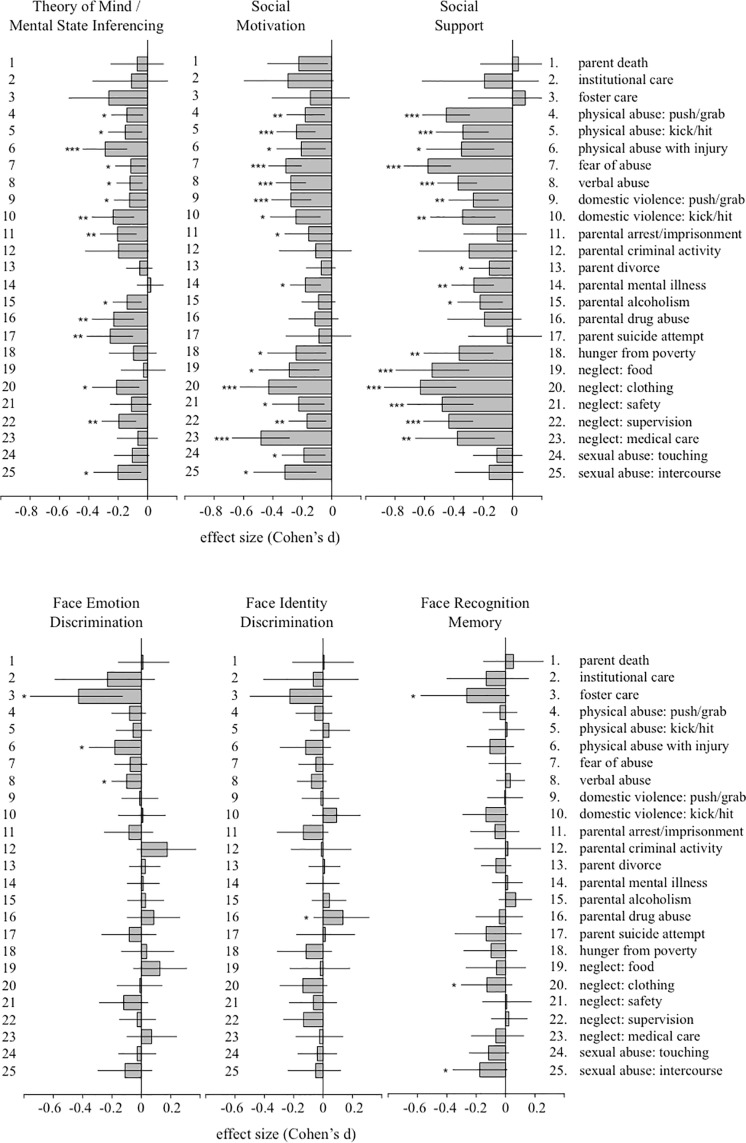
Associations between individual adversity exposure and social cognition / social affiliation measures. Results are given in terms of Cohen’s *d* by exposure (exposed versus non-exposed), controlling for influence of age, sex, and race/ethnicity on each dependent measure. Solid lines give 95% confidence intervals for Cohen’s d. Three asterisks indicate associations that were significant based on independent samples-t-test at p < 0.00033 (Bonferroni corrected for all comparisons), two asterisks indicate associations significant at p < 0.002 (Bonferroni corrected for number of adversities), and one asterisk indicates nominal significance at p < 0.05. As in the previous analysis, a number of childhood adversities robustly predicted theory of mind ability, social motivation, and social support, but none predicted face discrimination or face memory at above nominal levels of significance.

As a secondary analysis, we looked at the relationship between exposure to each childhood adversity, individually, and social cognition / social affiliation scores. This allowed us to address any specific associations that our principal components analysis may have obscured or omitted (e.g. parent death did not load highly on any individual component). Results for this analysis are given in Fig 4 in terms of Cohen’s *d*, based on the difference in scores among participants where the adversity was present vs. absent. Effect sizes are given with 95% confidence intervals, adjusting for age, sex, and race/ethnicity. Significance testing was done using independent sample t-tests, again with three levels of significance: nominal (p < 0.05 uncorrected), with Bonferonni correction for number of adversities (25 comparisons; p < 0.002), and with Bonferonni correction for number of adversities and outcome measures (25 x 6 = 150 comparisons; p < 0.00033). Effect size estimates and statistics for all individual adversity comparisons are given in S5 Table of supplementary materials.

Physical abuse was robustly associated with theory of mind ability, social motivation, and social support, with similar patterns of association for domestic violence. Aspects of parental neglect were strongly associated with social support and social motivation. Parental drug abuse, criminal behavior and suicide attempts were also associated with theory of mind ability. No individual adversity was associated with face recognition memory, face identity discrimination, or face emotion discrimination once any corrections for multiple comparisons were applied. We note that this pattern of associations cannot not be explained by differences in sensitivity or reliability among our social-cognitive measures (see Table 2).

## Discussion

In this study, we investigated the relationship between 25 common forms of childhood adversity and adult variations in social cognition and social affiliation. Among the four core social cognitive abilities assessed, theory of mind ability (or mental state inferencing ability) was the most clearly associated with experiences of childhood adversity, whereas face discrimination (emotion and identity) and face recognition memory showed no more than nominal associations with any form of childhood adversity in our samples. Childhood experiences of parental maltreatment (primarily physical abuse) and parental maladjustment (e.g. parental criminality, alcoholism, and drug abuse) were most strongly associated with theory of mind ability, although only parental maltreatment was also related to levels of social affiliation. Our results were essentially unaltered when differences in socioeconomic status were taken into account, suggesting that experiences of socioeconomic deprivation do not account for the observed associations. Putting these findings together, we propose that experiences of parental maltreatment such as physical abuse might lead to a reduced ability to accurately infer other people’s mental states and negatively impact a person’s ability to seek and maintain social ties. This reduction in social affiliation would mean a person receives less of the practical, psychological, and physiological benefits that come from interpersonal relationships [2].

Previous literature has linked severe forms of childhood adversity to differences in the way children process social and emotional information [3–8]. Our findings provide an important extension to this work by demonstrating that differences in social cognitive functioning related to childhood adversity are present (1) in adulthood, suggesting enduring effects of adverse childhood environments, and (2) for highly prevalent forms of adversity. Our findings highlight an important and lasting role for variations in early life stress on individual differences in adult social cognitive functioning. Future research might try to address whether differences in social cognitive functioning mediate the relationship between childhood adversity and adult mental health [10–12].

Our results highlight a dissociation within social cognition that indicates that the relationship between childhood adversity and adult cognitive functioning is somewhat domain-specific. The lack of association between childhood adversity and face recognition memory / face discrimination indicates that common forms of childhood adversity are not strongly associated with differences in generalized information processing, perceptual or social perceptual impairments. If such impairments did exist, we would expect these impairments to be reflected in reduced face memory and face discrimination abilities. Further research might help broaden this picture by looking at the relationship between childhood adversity and nonsocial cognitive abilities, in a similar epidemiological / individual differences framework. Our results suggest that the effects of common forms of childhood adversity do not generalize across domains of social cognitive ability. Our results are consistent with the view that face identity recognition is a highly specific and highly heritable domain of cognitive functioning [13], resilient to the impact of commonly occurring differences in environment.

Previous literature has implicated neglect as a predictor of broad differences in cognitive and social cognitive ability [3,6,7]. Contrary to our hypothesis, we found that neglect was only associated with self-reported levels of social affiliation and not objectively measured aspects of social cognition. We would infer these differences arise from differences in the form and severity of neglect that predominates in our unselected sample vs. studies that used targeted samples of neglected children identified either through institutions or social services in other studies. The forms of parental neglect we assessed are comparable to the types of parental neglect assessed in large-scale psychiatric epidemiology studies, where neglect has been found to be associated with adult mental health [10,11]. It may be that social cognitive differences do not contribute substantially to the associations between parental neglect and mental health identified in large population-based studies.

Our results support the “tuning” hypothesis of childhood adversity, which suggests that experiences of adversity like violence bias the way an individual evaluates social information [8,33]. Differences in the tuning of social judgments would impact the ability to make nuanced judgments of mental states, but not necessarily impact basic competencies that are less evaluation-driven—such as face identity processing or face discrimination. This is consistent with evidence from the literature on childhood physical abuse, which indicates that basic emotion recognition is not impaired *per se* in children who suffer from physical abuse [4]. Instead, abuse may alter a person’s sensitivity to certain types of social information, thus biasing judgments of mental state.

In this study, we implicitly assumed that experiences of childhood adversity impact the development of social cognition. It is possible, however, that adults with reduced social cognitive abilities may be more likely to create (or place their children in) adverse environments. In this case, the observed associations between childhood adversity and social cognition may arise from shared genetic factors that impact social cognitive development in parent and child. Although familial risk does not seem to account for the relationship between childhood physical abuse and certain psychiatric disorders [34], more work would be needed to address the role of genetic factors in the relationship between childhood adversity and social cognitive outcomes.

It is also possible that individuals with certain difficulties in social cognition were more likely to *recall* experiences of childhood adversity. Recent analyses indicate that the relationship between childhood adversity and adult mental illness (based on self-reported symptoms) is similar whether adversity is assessed retrospectively or prospectively [35]. It is also worth noting that prospective studies suffer from their own reporting biases related to willingness to disclose adversities that are occurring or have recently occurred (e.g. sexual abuse) [36]. Like any approach, our choice of methodology has strengths and weaknesses that make convergent findings from other types of study designs important for drawing conclusions about how adversity contributes to social cognitive development.

Finally, we note that average differences in social cognition and social affiliation between those had been exposed or not exposed to any particular adversity were much smaller than the differences that existed between individuals within those groups. In other words, group differences were small and observed in the context of wide variability in social cognitive ability and social affiliation within each group. Likewise, associations that were weak in our sample and did not survive correction for multiple comparisons (such as the relationship between face identity recognition ability and being in foster care as a child) may nevertheless reflect meaningful differences that could be detected with more targeted experimental designs. Indeed, we hope that our results serve as a foundation for generating new hypotheses about the way early life experience impacts our social development.

## Conclusions

In summary, our results indicate that experiences of childhood adversity are most strongly linked to differences in theory of mind ability, or mental state inferencing, as well as self-reported levels of social affiliation (social motivation and social support). Face discrimination and face memory abilities, on the other hand, appear to be relatively unaffected by early adversity. This is consistent with previous literature indicating dissociable mechanisms that underlie face discrimination and other aspects of social perception [37–39]. Our findings identify areas of social cognitive development that may be particularly sensitive to common variations in childhood environment, suggesting potential mechanisms linking childhood adversity and adult mental health through variations in the development of social cognitive functioning.

Finally, this study represents the first application of large-scale, web-based crowdsourcing methods for understanding the way childhood adversity is related to individual differences in cognition. This approach gave us a unique opportunity to explore the rich and multidimensional landscape of childhood environment and social cognitive development in a way that has not previously been practically feasible.

## Supporting Information

S1 Table Childhood Experiences Questionnaire. Shown above are items from the childhood experiences questionnaire administered to all participants. The questionnaire assesses 25 types of childhood adversity experience as well as information about childhood socioeconomic status. Questions about timing and duration of different adversities were included in the protocol, but analysis and reporting of these data are not included for the current manuscript. To see the exact formatting and organization of the questionnaire, as seen by participants, please go to http://testmybrain.org/tests/childhood_adversity.html
(DOC)

S2 Table Prevalence estimates for each childhood adversity in each subsample. Prevalence estimates reflect the proportion of adult participants who self-reported that they had been exposed to an adversity before the age of 18. A participant was considered exposed to an adversity if they answered “yes” to a question regarding whether a particular adversity occurred or “sometimes”/ “often” when asked about the frequency of a particular adversity experience. See S1 Table for specific items in the questionnaire.
(DOC)

S3 Table Loading of each adversity onto components derived from Principal Component Analysis (PCA). Four components of childhood adversity were derived by applying Principal Component Analysis (PCA) with varimax rotation to the entire sample of childhood adversity data. Component loadings less than 0.1 are not shown.
(DOC)

S4 Table The relationship between childhood adversity principal components and scores on each measure of social functioning. Linear regression was used to look at the relationship between PCA-derived childhood adversity components and social functioning across three different models. Model 1 estimated the relationship between childhood adversity components and social functioning without any covariates. Model 2 included sex, age, and race/ethnicity as covariates. Model 3 included three additional covariates reflecting different indices of socioeconomic status. Each table gives a regression coefficient estimate, standard error of estimate, t-value for each coefficient estimate, and p-value for each coefficient estimate. Scores on all social functioning measures were converted to z-scores. Sex was coded with male as 1 and female as 0. Age was not recoded. Race/ethnicity was coded with nonhispanic caucasian as 1 and all others as 0. Relative household income and parental education were coded by level ranging from 0 to 4, where higher numbers reflect greater household income and parental education.
(DOC)

S5 Table The relationship between individual childhood adversity experiences and residualized scores on each measure of social functioning. Shown are effect size estimates for participants who reported being exposed vs. not exposed to each adversity type. Effect sizes are given in terms of Cohen’s *d*, reflecting the mean difference between the two groups divided by the pooled standard deviation in scores. Lower and upper bounds for the 95% confidence interval around this effect size estimate are also shown, along with associated p-values. Confidence intervals and p-values were determined using bootstrap resampling procedures. Given a subsample *S* of size N_s_ corresponding to participants who completed a particular social functioning measure, we sampled with replacement N_s_ times from *S* and computed Cohen’s *d* for individuals exposed vs. not exposed to each childhood adversity type. This procedure was repeated 1000 times per subsample to generate standard error estimates. These standard errors were used to compute 95% confidence intervals around each estimate. Two-tailed independent samples *t*-tests were then applied to these mean and standard error estimates to generate *p*-values for each comparison. All comparisons were conducted with residualized social functioning scores, after variations due to age, sex, and race/ethnicity were removed.
(DOC)
