# Supplementary material for: Childhood Adversity Is Associated with Adult Theory of Mind and Social Affiliation, but Not Face Processing
Source: PLoS One. 2015 Jun 12;10(6):e0129612. doi: 10.1371/journal.pone.0129612 (PMC4466913; doi:10.1371/journal.pone.0129612)
Supplement: S1 Table — Shown above are items from the childhood experiences questionnaire administered to all participants. The questionnaire assesses 25 types of childhood adversity experience as well as information about childhood socioeconomic status. Questions about timing and duration of different adversities were included in the protocol, but analysis and reporting of these data are not included for the current manuscript. To see the exact formatting and organization of the questionnaire, as seen by participants, please go to http://testmybrain.org/tests/childhood_adversity.html (DOC) [file pone.0129612.s001.doc]

**Childhood Experiences Survey**

Please answer each of the following questions about experiences that may or may not have happened to you when you were a child or adolescent. Each question is about experiences you may have had **from the time you were born until you were 18 years old.**

1. What is the highest degree your *mother* earned?

<INPUT TYPE=\*High school diploma or certificate<INPUT TYPE=\*

*Bachelor's degree<INPUT TYPE=\*

*Master's degree<INPUT TYPE=\*

*Doctorate<INPUT TYPE=\*

*Professional (MD, JD, DDS, etc.)*

*Less than high school<INPUT TYPE=\*

*None of the above<INPUT TYPE=\*

*I don't know*

*<INPUT TYPE=\I'd rather not say*

1. What is the highest degree your *father* earned?

*High school diploma or certificate*

*Bachelor's degree<INPUT TYPE=\*

*Master's degree*

*Doctorate<INPUT TYPE=\*

*Professional (MD, JD, DDS, etc.)*

*<INPUT TYPE=\Less than high school<INPUT TYPE=\*

*None of the above*

*I don't know*

*<INPUT TYPE=\I'd rather not say*

1. Compared to the other families in the same country or part of the country, what was your family's income?

<INPUT TYPE=\*Much lower than the average household<INPUT TYPE=\*

*Somewhat lower than the average household*

*Similar to the average household<INPUT TYPE=\*

*Somewhat higher than the average household<INPUT TYPE=\*

*Much higher than the average household*

*<INPUT TYPE=\I don't know*

*<INPUT TYPE=\I'd rather not say*

1. When you were a child or adolescent, did either of your parents die?

<INPUT TYPE=\*Yes /<INPUT TYPE=\ No<INPUT TYPE=\ / I don't know<INPUT TYPE=\ / I'd rather not say*

1. If yes, how old were you when this happened?
2. When you were a child or adolescent, did your parents ever separate or get divorced?

<INPUT TYPE=\ <INPUT TYPE=\ *<INPUT TYPE=\Yes / No / I don’t know / I’d rather not say*

1. If yes, how old were you when this happened?
2. When you were a child or adolescent, were you ever placed in foster care?

*<INPUT TYPE=\Yes / No / I don’t know / I’d rather not say*

1. If yes, how old were you when this happened?

<SELECT NAME=\

1. If yes, what was the total amount of time you spent in foster care?

<SELECT NAME=\

1. When you were a child or adolescent, did you ever live in a children's home or an institution?

<INPUT TYPE=\ <INPUT TYPE=\ *<INPUT TYPE=\Yes / No / I don’t know / I’d rather not say*

1. If yes, how old were you when this happened?

<SELECT NAME=\

1. If yes, what was the total amount of time you spent in the institution?

<SELECT NAME=\

1. When you were a child or adolescent, was either of your parents or another caregiver a problem drinker or alcoholic?

<INPUT TYPE=\ <INPUT TYPE=\ *<INPUT TYPE=\Yes / No / I don’t know / I’d rather not say*

1. When you were a child or adolescent, did either of your parents or another caregiver regularly use street drugs?

<INPUT TYPE=\ <INPUT TYPE=\ *<INPUT TYPE=\Yes / No / I don’t know / I’d rather not say*

1. When you were a child or adolescent, was either of your parents or another caregiver depressed or mentally ill?

*<INPUT TYPE=\Yes / No / I don’t know / I’d rather not say*

1. When you were a child or adolescent, did either of your parents or another caregiver ever attempt suicide?

<INPUT TYPE=\ <INPUT TYPE=\ *<INPUT TYPE=\Yes / No / I don’t know / I’d rather not say*

1. If yes, how old were you when this happened?

<SELECT NAME=\

1. When you were a child or adolescent, was either of your parents or another caregiver ever arrested or sent to prison?

<INPUT TYPE=\ <INPUT TYPE=\ *<INPUT TYPE=\Yes / No / I don’t know / I’d rather not say*

1. If yes, how old were you when this happened?
2. When you were a child or adolescent, was either of your parents or another caregiver involved in criminal activity like burglary or selling stolen property?

*Often / Sometimes / Rarely / Never / I don't know / I'd rather not say*

1. When you were a child or adolescent, did your family receive money from a government assistance program? In the U.S., this includes programs like welfare, food stamps, Aid to Families with Dependent Children, or Temporary Assistance for Needy Families?

*Often / Sometimes / Rarely / Never / I don't know / I'd rather not say*

1. If yes, about how many years between the time you were born and the time you turned 18 did your family receive money from a government assistance program?
2. When you were a child or adolescent, how often were you hungry but either couldn't eat or ate less than you wanted because your family did not have enough money to buy food?

*Often / Sometimes / Rarely / Never / I don't know / I'd rather not say*

1. When you were a child or adolescent, how often did a parent or other caregiver swear at you, insult you, or put you down?

<INPUT TYPE=\*Often / Sometimes / Rarely / Never / I don't know / I'd rather not say*

1. If yes, how old were you **the first time** this happened?

<SELECT NAME=\

1. If yes, how old were you **the last time** this happened?

<SELECT NAME=\

1. When you were a child or adolescent, how often did a parent or other caregiver make you fear that you might be physically hurt?

*Often / Sometimes / Rarely / Never / I don't know / I'd rather not say*

1. If yes, how old were you **the first time** this happened?

<SELECT NAME=\

1. If yes, how old were you **the last time** this happened?
2. When you were a child or adolescent, how often did a parent or other caregiver push, grab, or throw something at you?

*Often / Sometimes / Rarely / Never / I don't know / I'd rather not say*

1. If yes, how old were you **the first time** this happened?

<SELECT NAME=\

1. If yes, how old were you **the last time** this happened?

<SELECT NAME=\

1. When you were a child or adolescent, how often did a parent or other caregiver kick, bite, or hit you?

*Often / Sometimes / Rarely / Never / I don't know / I'd rather not say*

1. If yes, how old were you **the first time** this happened?

<SELECT NAME=\

1. If yes, how old were you **the last time** this happened?
2. When you were a child or adolescent, how often did a parent or other caregiver hit you so hard that you had bruises, marks or were injured?

*Often / Sometimes / Rarely / Never / I don't know / I'd rather not say*

1. If yes, how old were you **the first time** this happened?

<SELECT NAME=\

1. If yes, how old were you **the last time** this happened?
2. When you were a child or adolescent, did anyone in your household or another adult touch or fondle you in a sexual way or make you touch their body in a sexual way?

<INPUT TYPE=\ *<INPUT TYPE=\Yes / No / I don’t know / I’d rather not say*

1. If yes, how old were you **the first time** this happened?

<SELECT NAME=\

1. If yes, how old were you **the last time** this happened?

<SELECT NAME=\

1. If yes, how many times did this happen?

<INPUT TYPE=\

1. If yes, who was the person who touched you in a sexual way or made you touch them in a sexual way?

<INPUT TYPE=\*<INPUT TYPE=\Relative in my household<INPUT TYPE=\*

*Relative outside my household<INPUT TYPE=\*

*Unrelated adult<INPUT TYPE=\*

*I don't know<INPUT TYPE=\*

*I'd rather not say*

1. When you were a child or adolescent, did anyone in your household or another adult attempt to have or actually have oral, anal, or vaginal intercourse with you?

*<INPUT TYPE=\Yes / No / I don’t know / I’d rather not say*

1. If yes, how old were you **the first time** this happened?

<SELECT NAME=\

1. If yes, how old were you **the last time** this happened?

<SELECT NAME=\

1. If yes, how many times did this happen?

<INPUT TYPE=\

1. If yes, who was the person who touched you in a sexual way or made you touch them in a sexual way?

*<INPUT TYPE=\Relative in my household<INPUT TYPE=\*

*Relative outside my household*

*Unrelated adult<INPUT TYPE=\*

*I don't know<INPUT TYPE=\*

*I'd rather not say*

1. When you were a child or adolescent, how often were you made to do chores that were too difficult or dangerous for someone your age?

*Often / Sometimes / Rarely / Never / I don't know / I'd rather not say*

1. When you were a child or adolescent, how often were you left alone or unsupervised when you were too young to be alone?

*Often / Sometimes / Rarely / Never / I don't know / I'd rather not say*

1. When you were a child or adolescent, how often did you go without things you needed like clothes, shoes, or school supplies because your parents or caregivers spent the money on themselves?

*Often / Sometimes / Rarely / Never / I don't know / I'd rather not say*

1. When you were a child or adolescent, how often did you go hungry because your parents did not prepare regular meals?

*Often / Sometimes / Rarely / Never / I don't know / I'd rather not say*

1. When you were a child or adolescent, how often did your parents or caregivers ignore or fail to get you medical treatment when you were sick or hurt?

*Often / Sometimes / Rarely / Never / I don't know / I'd rather not say*

1. When you were a child or adolescent, how often did your parents or other caregivers push, grab, or throw something at each other?

*<INPUT TYPE=\<INPUT TYPE=\Often<INPUT TYPE=\ / Sometimes<INPUT TYPE=\ / Rarely<INPUT TYPE=\ / Never<INPUT TYPE=\ / I don't know<INPUT TYPE=\ / I'd rather not say*

1. If yes, how old were you **the first time** this happened?

<SELECT NAME=\

1. If yes, how old were you **the last time** this happened?
2. When you were a child or adolescent, how often did your parents or other caregivers kick, bite, or hit each other?

*<INPUT TYPE=\<INPUT TYPE=\Often<INPUT TYPE=\ / Sometimes<INPUT TYPE=\ / Rarely<INPUT TYPE=\ / Never<INPUT TYPE=\ / I don't know<INPUT TYPE=\ / I'd rather not say*

1. If yes, how old were you **the first time** this happened?

<SELECT NAME=\

1. If yes, how old were you **the last time** this happened?
2. Is there anything else you would like us to know?

**Table S1. Childhood Experiences Questionnaire**

Shown above are items from the childhood experiences questionnaire administered to all participants. The questionnaire assesses 25 types of childhood adversity experience as well as information about childhood socioeconomic status. Questions about timing and duration of different adversities were included in the protocol, but analysis and reporting of these data are not included for the current manuscript. To see the exact formatting and organization of the questionnaire, as seen by participants, please go to

<http://testmybrain.org/tests/childhood_adversity.html>
