# Supplementary material for: Childhood Adversity Is Associated with Adult Theory of Mind and Social Affiliation, but Not Face Processing
Source: PLoS One. 2015 Jun 12;10(6):e0129612. doi: 10.1371/journal.pone.0129612 (PMC4466913; doi:10.1371/journal.pone.0129612)
Supplement: S2 Table — Prevalence estimates reflect the proportion of adult participants who self-reported that they had been exposed to an adversity before the age of 18. A participant was considered exposed to an adversity if they answered “yes” to a question regarding whether a particular adversity occurred or “sometimes”/ “often” when asked about the frequency of a particular adversity experience. See S1 Table for specific items in the questionnaire. (DOC) [file pone.0129612.s002.doc]

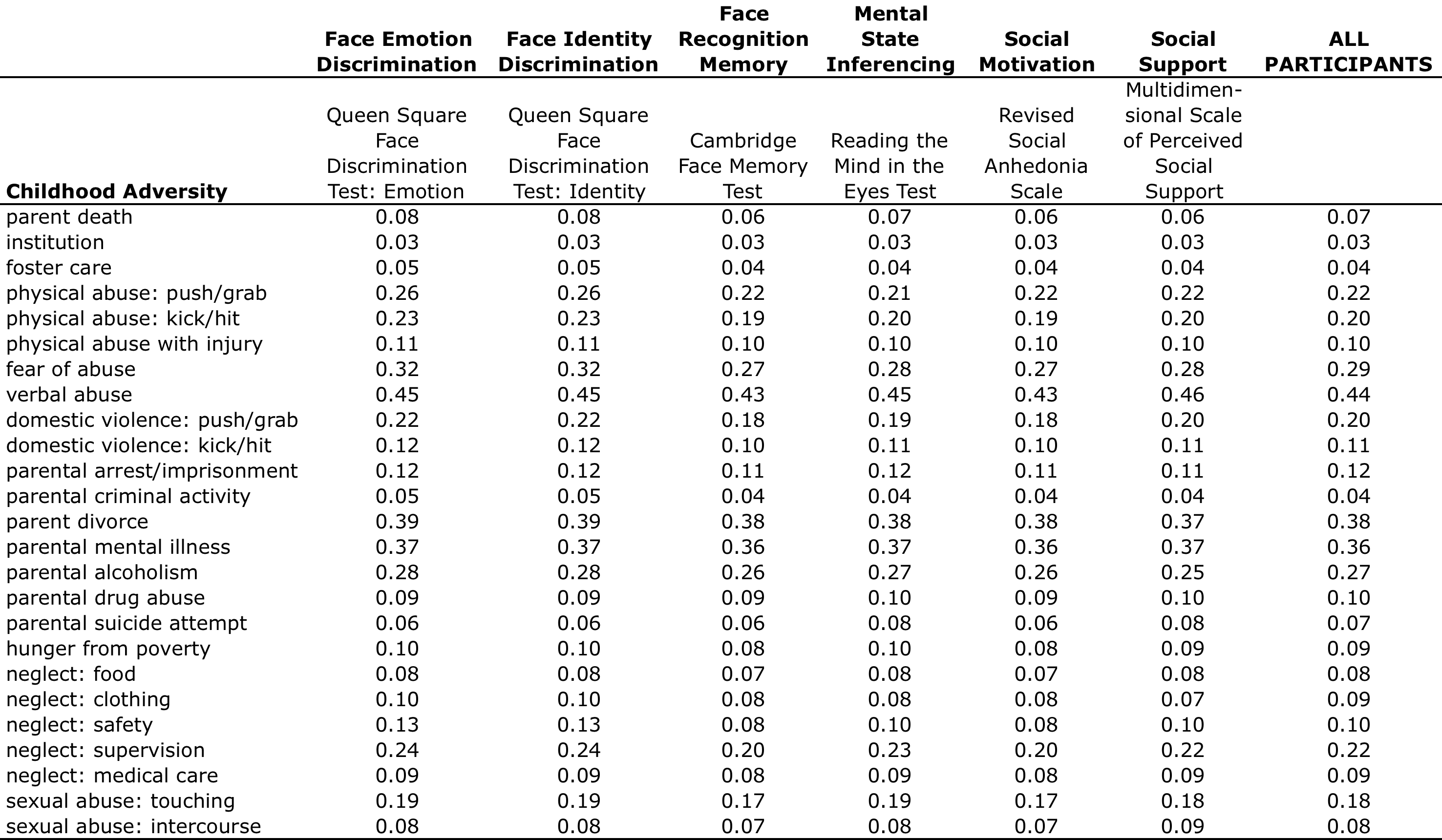
**Table S2. Prevalence estimates for each childhood adversity in each subsample**

Prevalence estimates reflect the proportion of adult participants who self-reported that they had been exposed to an adversity before the age of 18. A participant was considered exposed to an adversity if they answered “yes” to a question regarding whether a particular adversity occurred or “sometimes”/ “often” when asked about the frequency of a particular adversity experience. See Table S1 for specific items in the questionnaire.
