# Supplementary material for: Childhood Adversity Is Associated with Adult Theory of Mind and Social Affiliation, but Not Face Processing
Source: PLoS One. 2015 Jun 12;10(6):e0129612. doi: 10.1371/journal.pone.0129612 (PMC4466913; doi:10.1371/journal.pone.0129612)
Supplement: S3 Table — Four components of childhood adversity were derived by applying Principal Component Analysis (PCA) with varimax rotation to the entire sample of childhood adversity data. Component loadings less than 0.1 are not shown. (DOC) [file pone.0129612.s003.doc]

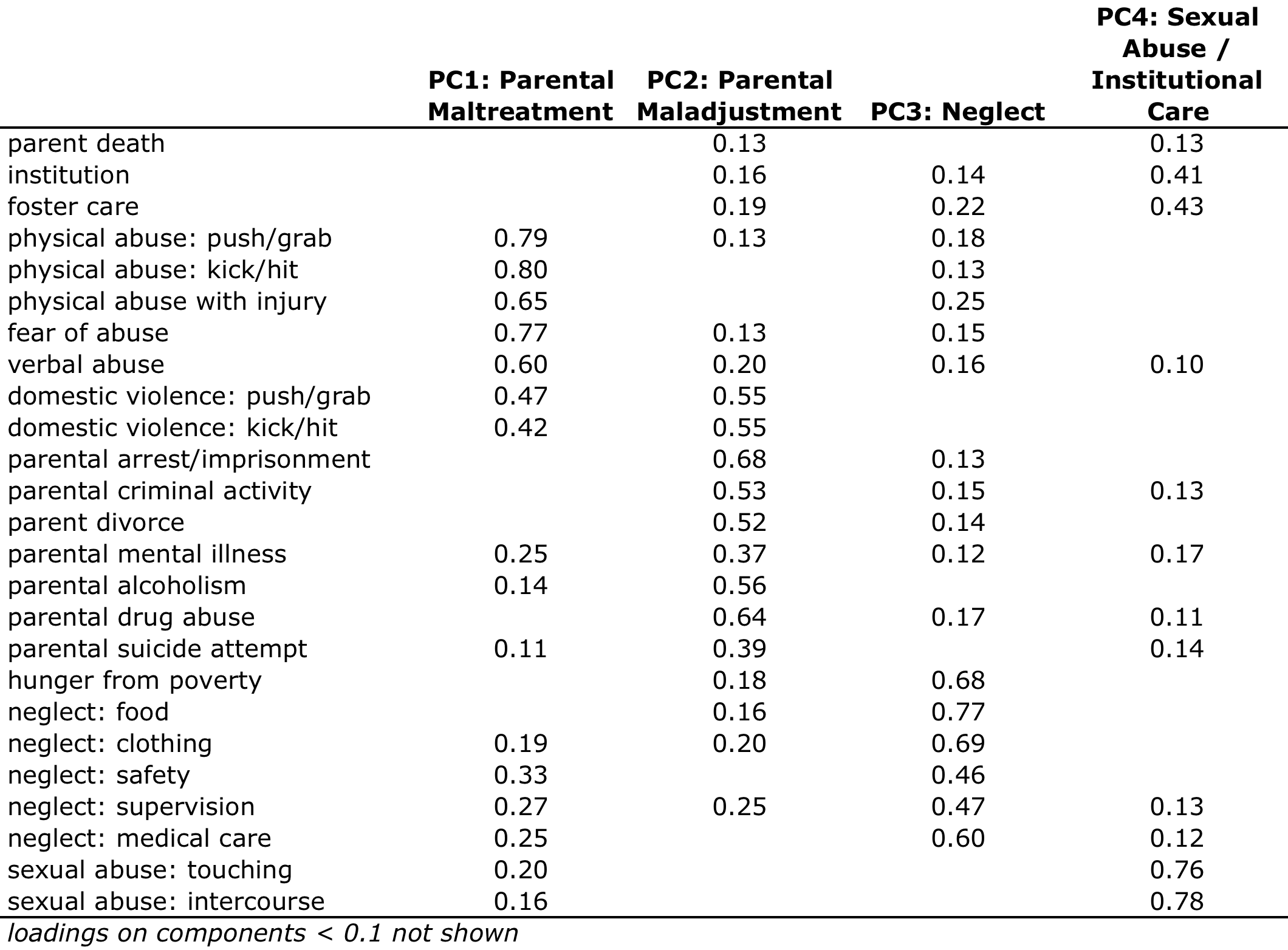
**Table S3: Loading of each adversity onto components derived from Principal Component Analysis (PCA)**

Four components of childhood adversity were derived by applying Principal Component Analysis (PCA) with varimax rotation to the entire sample of childhood adversity data. Component loadings less than 0.1 are not shown.
