# Supplementary material for: Childhood Adversity Is Associated with Adult Theory of Mind and Social Affiliation, but Not Face Processing
Source: PLoS One. 2015 Jun 12;10(6):e0129612. doi: 10.1371/journal.pone.0129612 (PMC4466913; doi:10.1371/journal.pone.0129612)
Supplement: S4 Table — Linear regression was used to look at the relationship between PCA-derived childhood adversity components and social functioning across three different models. Model 1 estimated the relationship between childhood adversity components and social functioning without any covariates. Model 2 included sex, age, and race/ethnicity as covariates. Model 3 included three additional covariates reflecting different indices of socioeconomic status. Each table gives a regression coefficient estimate, standard error of estimate, t-value for each coefficient estimate, and p-value for each coefficient estimate. Scores on all social functioning measures were converted to z-scores. Sex was coded with male as 1 and female as 0. Age was not recoded. Race/ethnicity was coded with nonhispanic caucasian as 1 and all others as 0. Relative household income and parental education were coded by level ranging from 0 to 4, where higher numbers reflect greater household income and parental education. (DOC) [file pone.0129612.s004.doc]

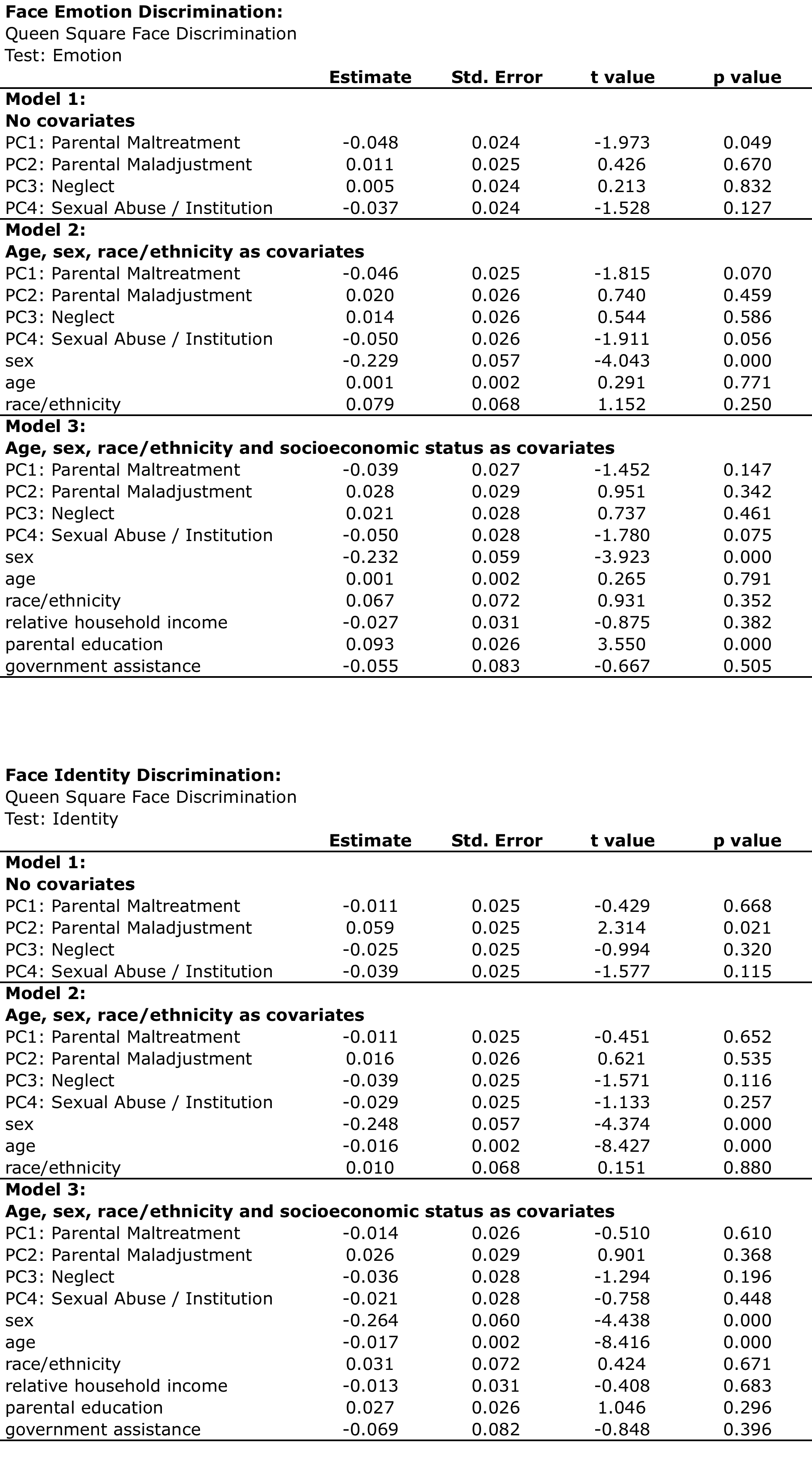


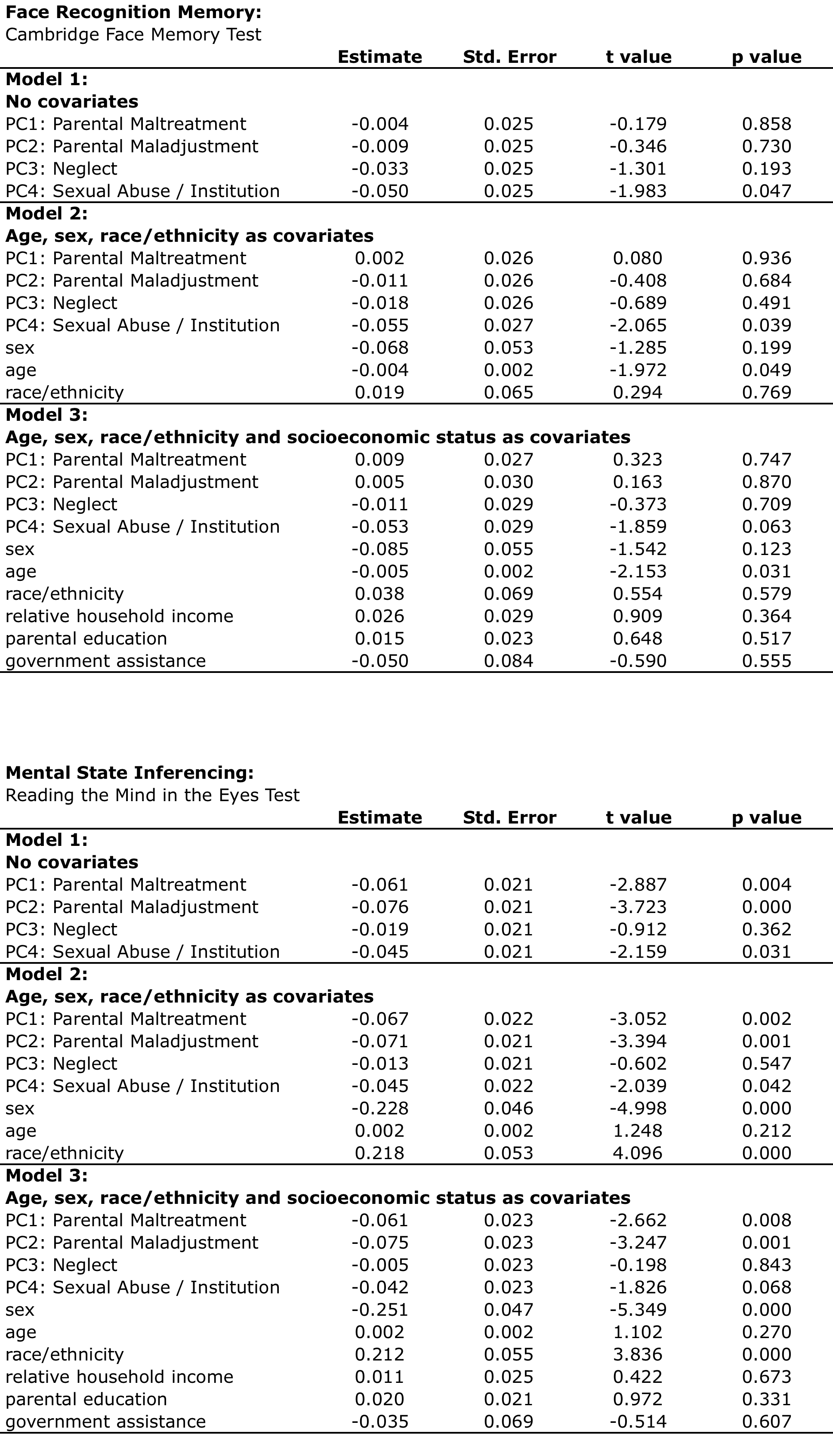


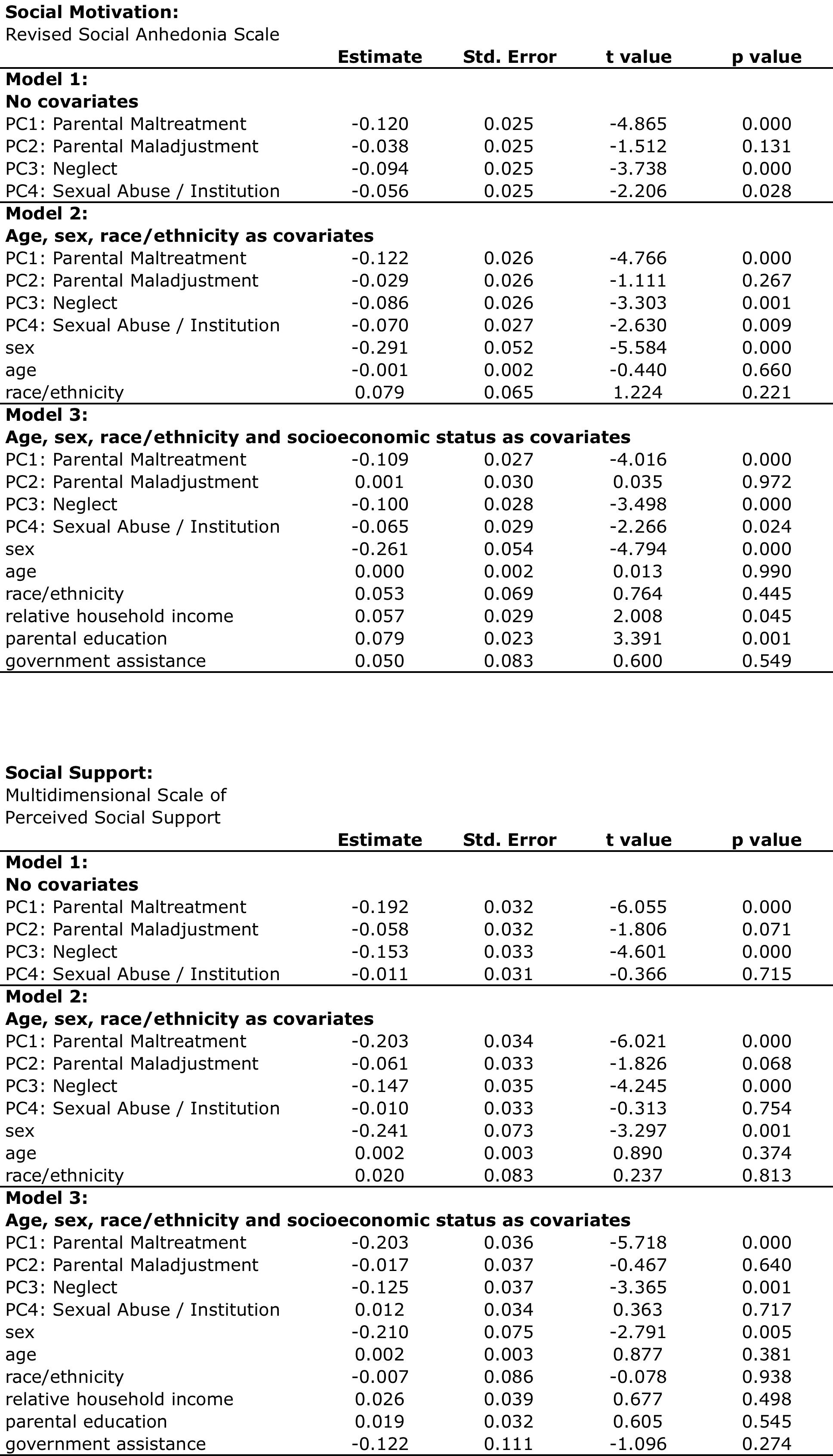


**Table S4. The relationship between childhood adversity principal components and scores on each measure of social functioning**

Linear regression was used to look at the relationship between PCA-derived childhood adversity components and social functioning across three different models. Model 1 estimated the relationship between childhood adversity components and social functioning without any covariates. Model 2 included sex, age, and race/ethnicity as covariates. Model 3 included three additional covariates reflecting different indices of socioeconomic status. Each table gives a regression coefficient estimate, standard error of estimate, t-value for each coefficient estimate, and p-value for each coefficient estimate. Scores on all social functioning measures were converted to z-scores. Sex was coded with male as 1 and female as 0. Age was not recoded. Race/ethnicity was coded with nonhispanic caucasian as 1 and all others as 0. Relative household income and parental education were coded by level ranging from 0 to 4, where higher numbers reflect greater household income and parental education.
