# Supplementary material for: Childhood Adversity Is Associated with Adult Theory of Mind and Social Affiliation, but Not Face Processing
Source: PLoS One. 2015 Jun 12;10(6):e0129612. doi: 10.1371/journal.pone.0129612 (PMC4466913; doi:10.1371/journal.pone.0129612)
Supplement: S5 Table — Shown are effect size estimates for participants who reported being exposed vs. not exposed to each adversity type. Effect sizes are given in terms of Cohen’s d, reflecting the mean difference between the two groups divided by the pooled standard deviation in scores. Lower and upper bounds for the 95% confidence interval around this effect size estimate are also shown, along with associated p-values. Confidence intervals and p-values were determined using bootstrap resampling procedures. Given a subsample S of size Ns corresponding to participants who completed a particular social functioning measure, we sampled with replacement Ns times from S and computed Cohen’s d for individuals exposed vs. not exposed to each childhood adversity type. This procedure was repeated 1000 times per subsample to generate standard error estimates. These standard errors were used to compute 95% confidence intervals around each estimate. Two-tailed independent samples t-tests were then applied to these mean and standard error estimates to generate p-values for each comparison. All comparisons were conducted with residualized social functioning scores, after variations due to age, sex, and race/ethnicity were removed. (DOC) [file pone.0129612.s005.doc]

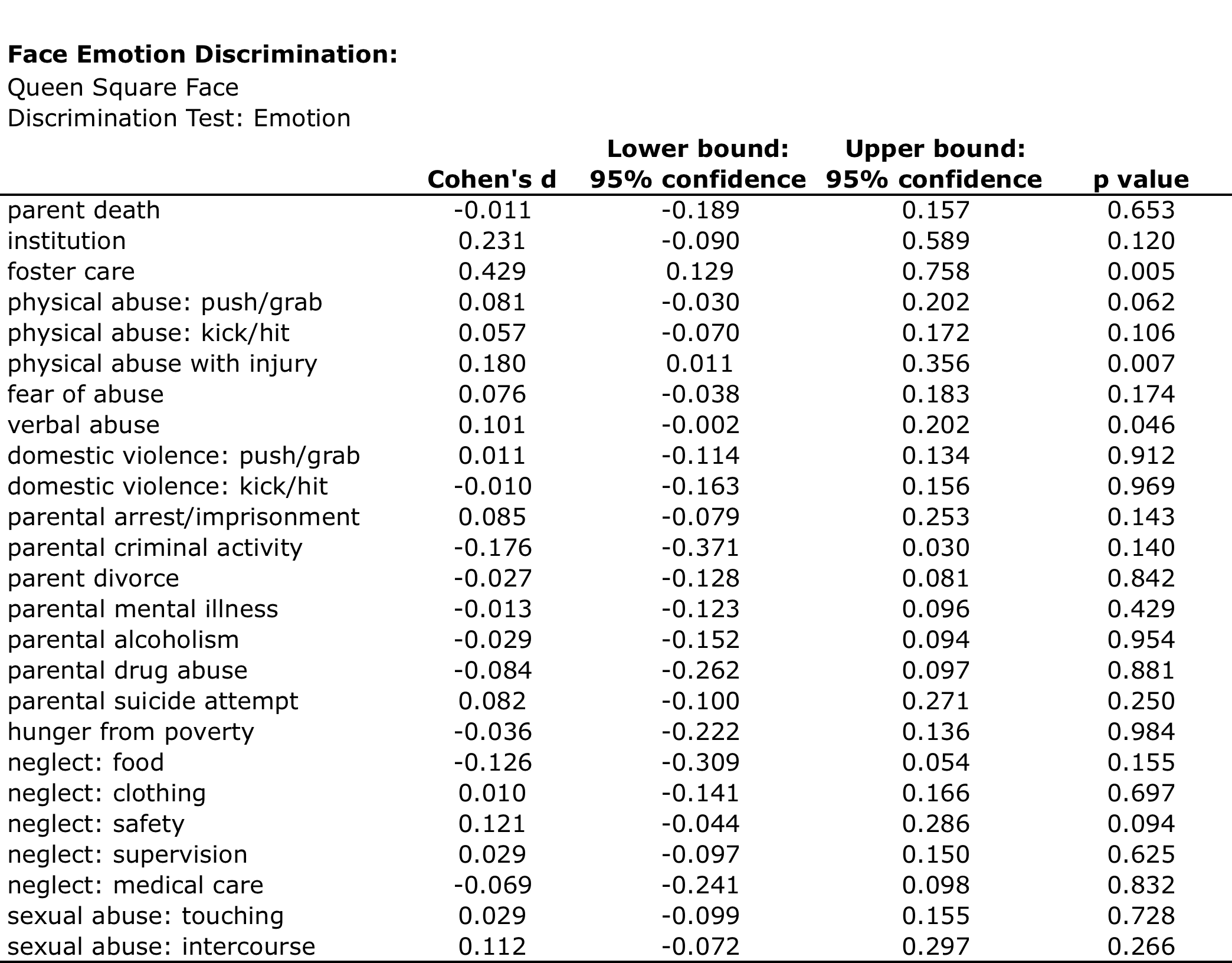

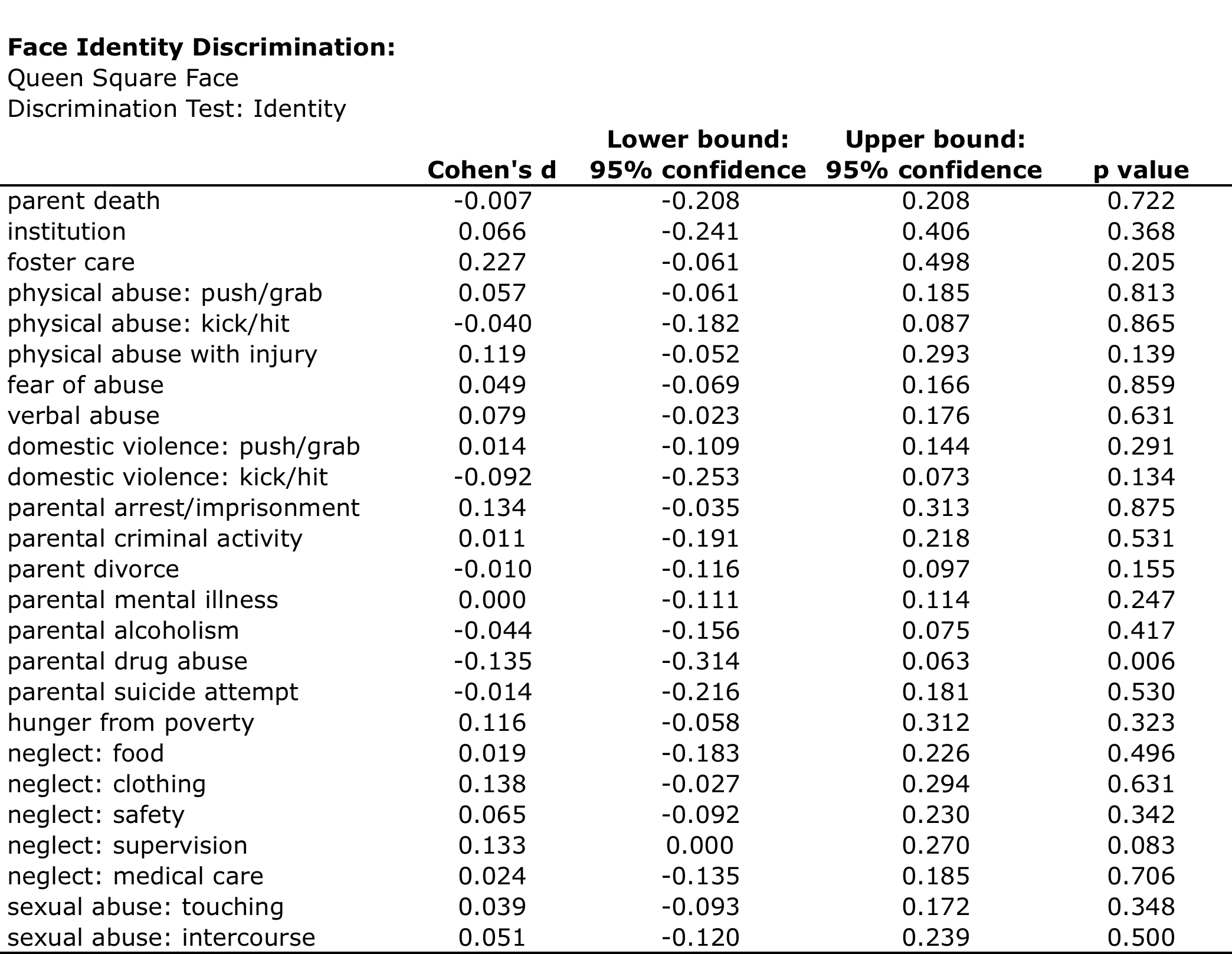


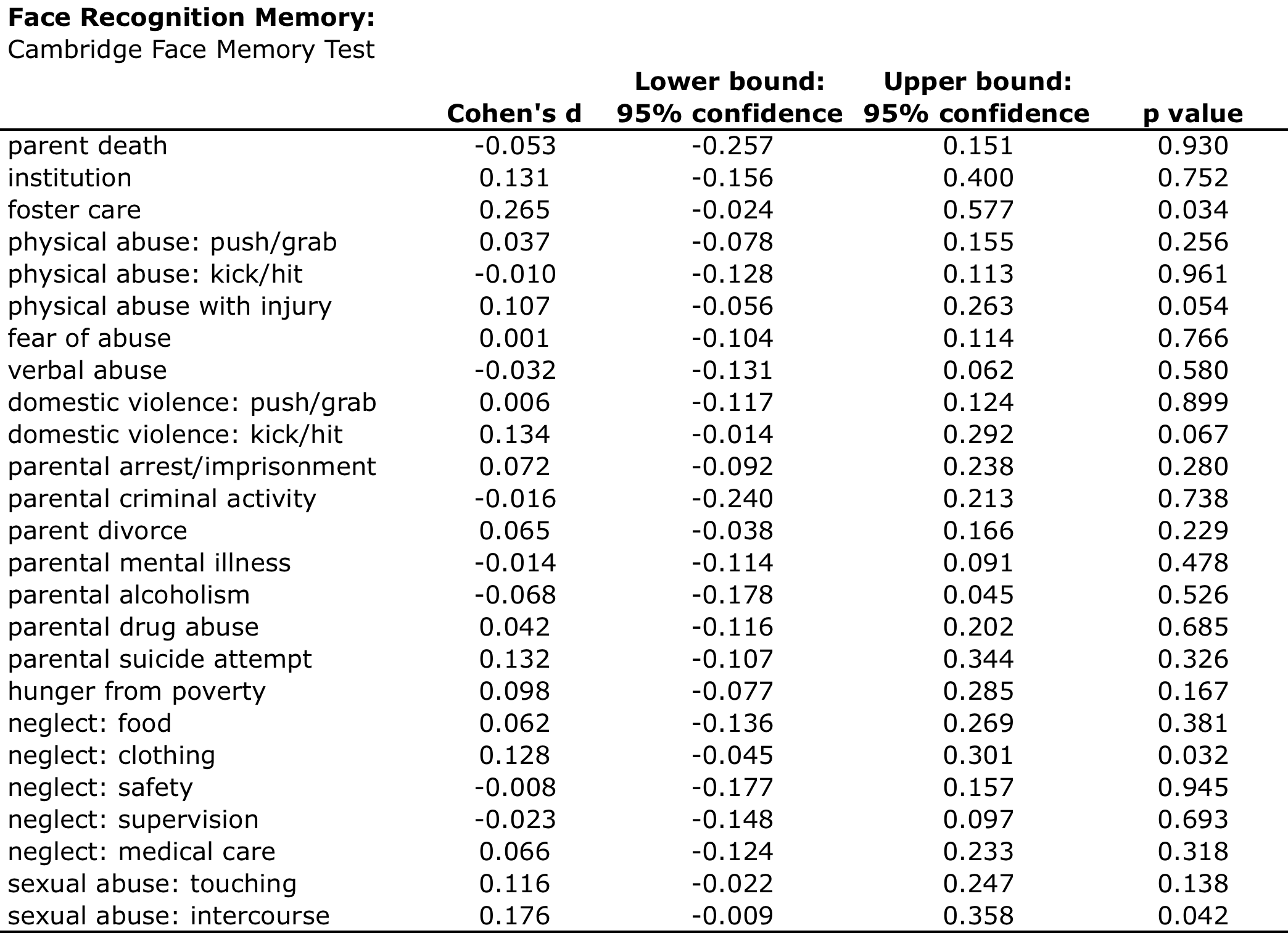

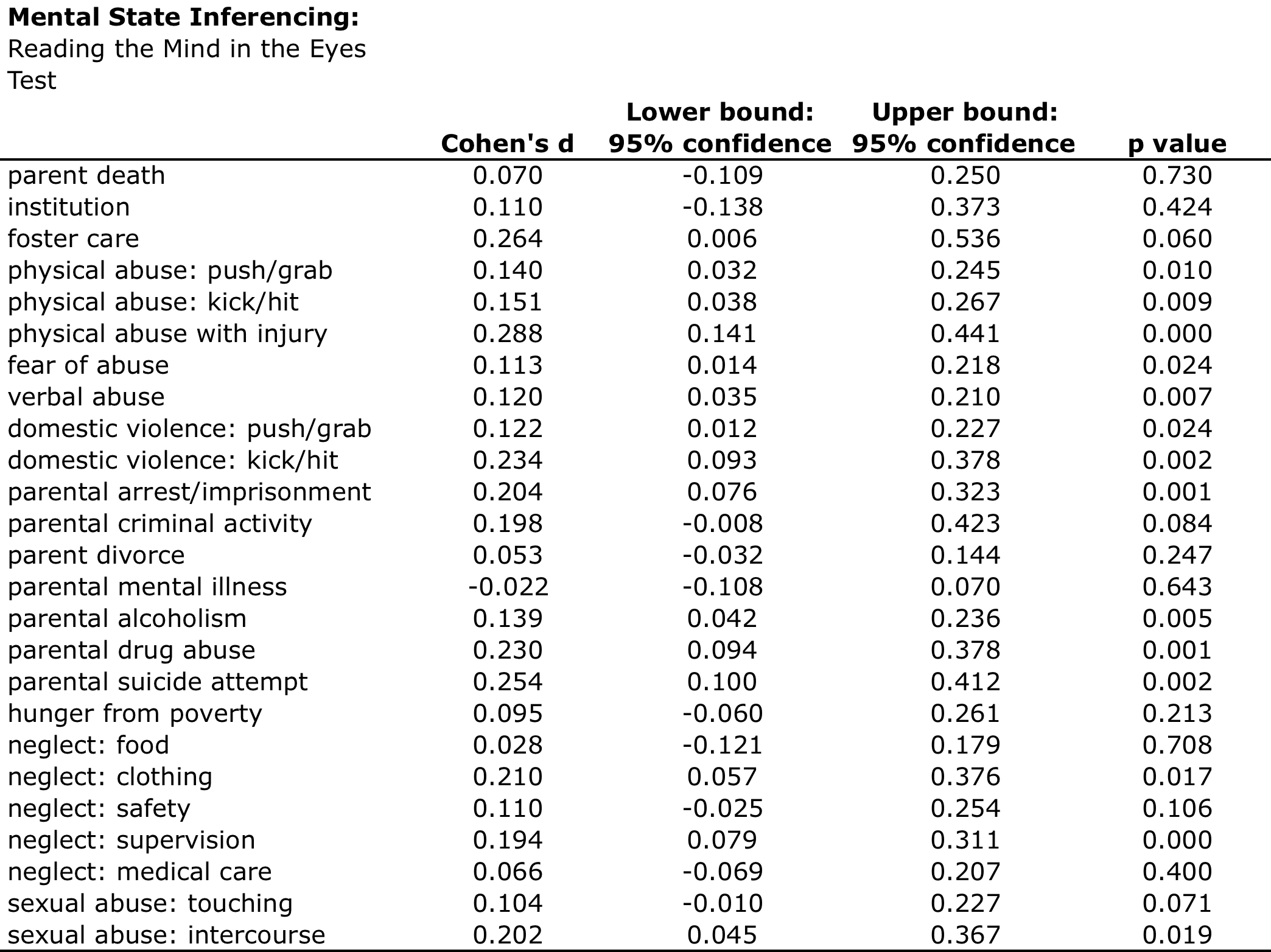


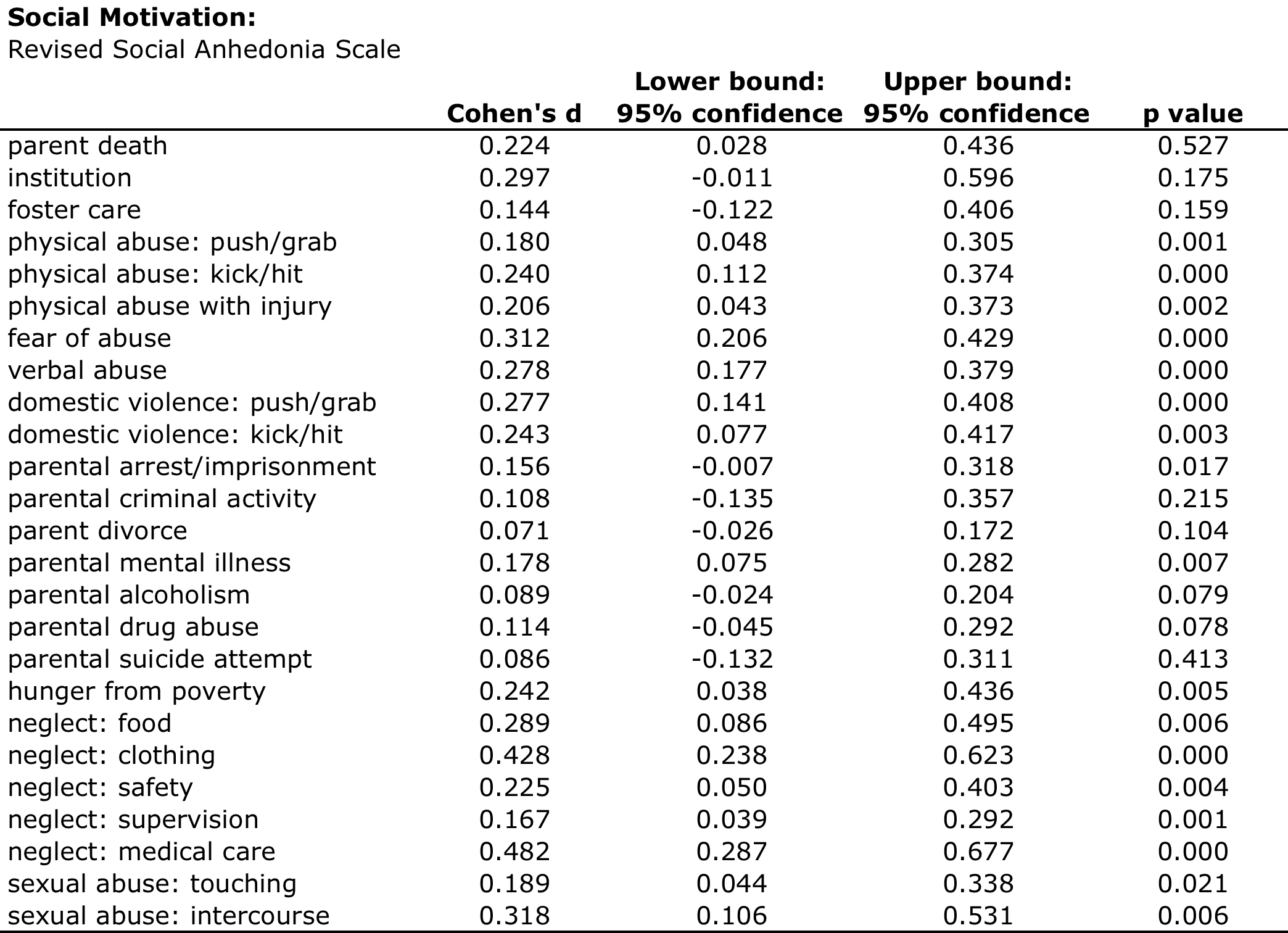

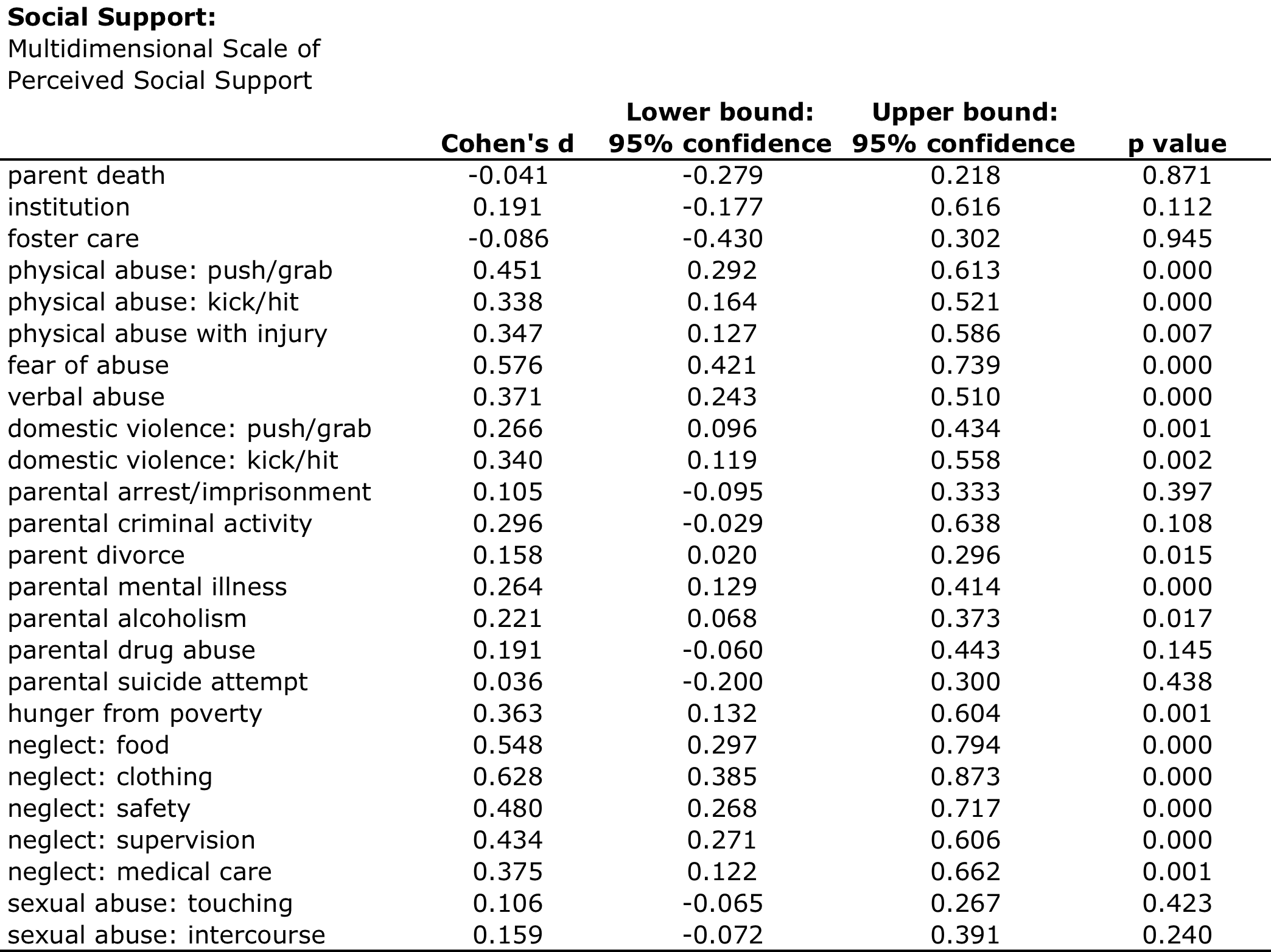


**Table S5. The relationship between individual childhood adversity experiences and residualized scores on each measure of social functioning**

Shown are effect size estimates for participants who reported being exposed vs. not exposed to each adversity type. Effect sizes are given in terms of Cohen’s *d*, reflecting the mean difference between the two groups divided by the pooled standard deviation in scores. Lower and upper bounds for the 95% confidence interval around this effect size estimate are also shown, along with associated p-values. Confidence intervals and p-values were determined using bootstrap resampling procedures. Given a subsample *S* of size N_s_ corresponding to participants who completed a particular social functioning measure, we sampled with replacement N_s_ times from *S* and computed Cohen’s *d* for individuals exposed vs. not exposed to each childhood adversity type. This procedure was repeated 1000 times per subsample to generate standard error estimates. These standard errors were used to compute 95% confidence intervals around each estimate. Two-tailed independent samples *t*-tests were then applied to these mean and standard error estimates to generate *p*-values for each comparison. All comparisons were conducted with residualized social functioning scores, after variations due to age, sex, and race/ethnicity were removed.
